# Novel DNA targeted therapies for head and neck cancers: clinical potential and biomarkers

**DOI:** 10.18632/oncotarget.20953

**Published:** 2017-09-16

**Authors:** Mary Glorieux, Rüveyda Dok, Sandra Nuyts

**Affiliations:** ^1^ KU Leuven, University of Leuven, Department of Oncology, Laboratory of Experimental Radiotherapy, 3000 Leuven, Belgium; ^2^ Department of Radiation Oncology, Leuven Cancer Institute, UZ Leuven, 3000 Leuven, Belgium

**Keywords:** HNSCC, DNA targeted agents, radiation sensitization, biomarkers

## Abstract

Head and neck squamous cell carcinoma is the sixth most common cancer worldwide and despite advances in treatment over the last years, there is still a relapse rate of 50%. New therapeutic agents are awaited to increase the survival of patients. DNA repair targeted agents in combination with standard DNA damaging therapies are a recent evolution in cancer treatment. These agents focus on the DNA damage repair pathways in cancer cells, which are often involved in therapeutic resistance. Interesting targets to overcome these cancer defense mechanisms are: PARP, DNA-PK, PI3K, ATM, ATR, CHK1/2, and WEE1 inhibitors. The application of DNA targeted agents in head and neck squamous cell cancer showed promising preclinical results which are translated to multiple ongoing clinical trials, although no FDA approval has emerged yet. Biomarkers are necessary to select the patients that can benefit the most from this treatment, although adequate biomarkers are limited and validation is needed to predict therapeutic response.

## INTRODUCTION

Head and neck squamous cell carcinoma (HNSCC) is the sixth most common cancer worldwide, resulting in approximately 550,000 diagnoses and 300,000 deaths a year [1]. Risk factors for HNSCC include tobacco and alcohol use, involved in 75% of the cases, and infection with human papilloma virus (HPV), associated with 40–60% of the oropharyngeal cancers [2, 3]. Standard treatment for HNSCC is a combination of surgery, radiotherapy (RT) and chemotherapy (CT) as most HNSCC are locally advanced at time of diagnosis. [4]. The five year survival rate of 40–50% is relatively poor despite advances in surgical techniques, chemo- and radiotherapy (cRT) [5]. Unfortunately, there is an inability to further intensify the current therapy due to unacceptable toxicity and morbidity [2]. Taking into account the high relapse rate and the limited therapeutic options, it is of utmost importance to underpin the molecular mechanisms of resistance.

**Figure 1:**
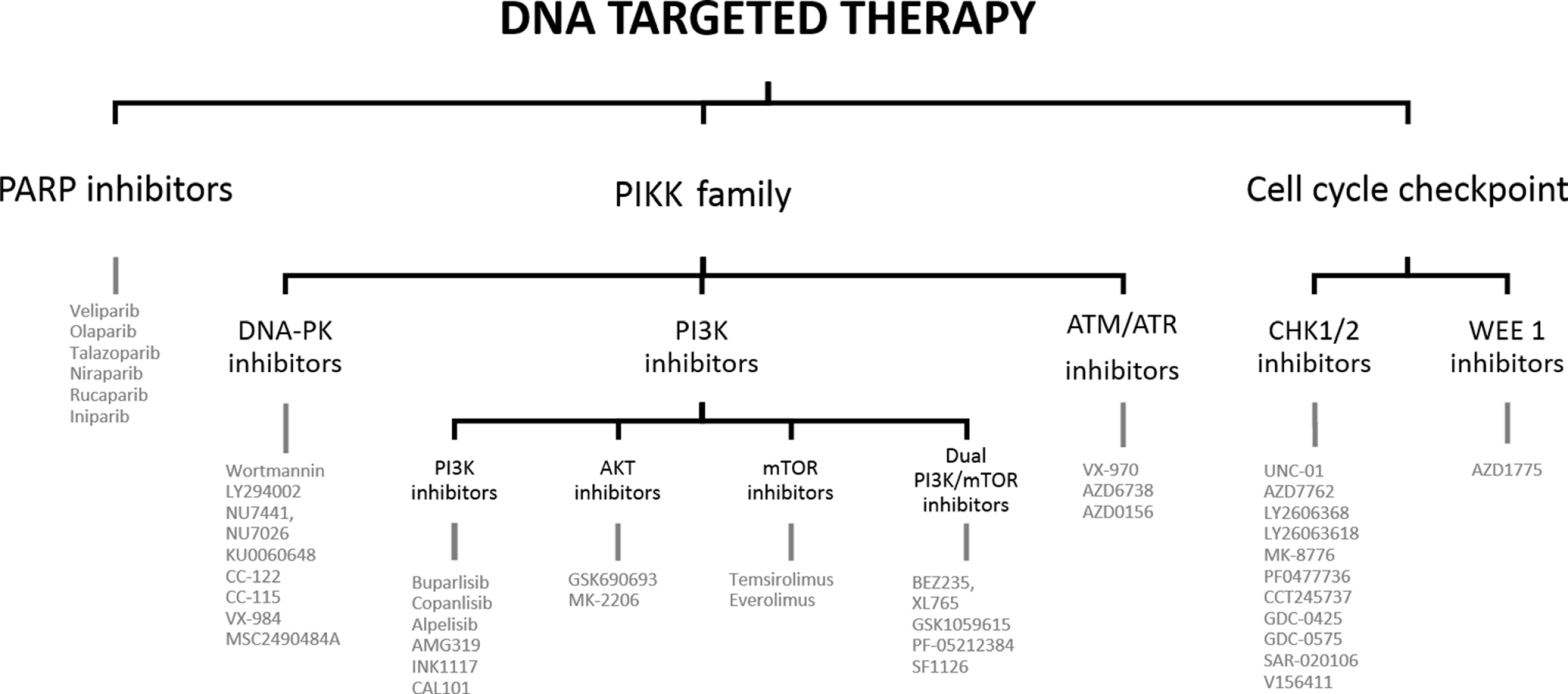
Outline of the review

The significant local relapse rate in HNSCC is mainly due to the high DNA repair capacity of cancer cells. Cells rely on the DNA damage response (DDR) to signal the presence of DNA damage so this can be repaired in order to survive. The key DNA repair pathways are: base excision repair (BER), nucleotide excision repair (NER) and mismatch repair (MMR) for single strand DNA breaks (SSBs), and homologous recombination (HR) and non-homologous end joining (NHEJ) for double strand breaks (DSBs). The exact mechanism of these pathways is beyond the scope of this review, but are clearly explained in the following papers [6–9]. Cancer cells often have deficiencies in these DNA repair pathways enabling the tumor cells to accumulate genetic alterations which attribute to their aggressive phenotype [9]. On the other hand, cancer cells rely on the remaining proficient DNA repair pathways to survive. DNA targeted agents try to exploit these backup DNA repair processes to generate synthetic lethality [8]. Cetuximab, a recombinant monoclonal antibody against the epidermal growth factor receptor (EGFR), is the only targeted agent for HNSCC to date that was approved by the Federal Food and Drug Administration (FDA). Because of the high resistance and low response rates, the search for suitable alternatives is urging. Modulating DNA repair after cRT with targeted agents is a promising technique to increase the therapeutic efficacy and decrease normal tissue toxicity. This can benefit a large number of HNSCC patients and is even applicable for a wide range of tumors [7]. In this review several possible DNA targeted therapies for HNSCC are discussed (see Figure 1).

## PARP INHIBITORS

Poly ADP-ribose polymerase (PARP) enzymes have a variety of important functions: DNA damage repair, cell cycle regulation and replication which influence tumor growth and progression [10–12]. PARP1 is an important sensor for SSBs and plays a critical role in BER [9, 13]. When DDR is activated, PARP1 synthetizes PAR polymers via autoPARylation to reseal and repair the DNA damage [14]. Recently it has been shown that PARP1 is also a regulator of HR, that it is involved in the alternative NHEJ and is required for restarting stalled/collapsed replication forks [9, 14–16]. Therefore PARP inhibition is interesting for both the regulation of transcription and inhibiting its function in DNA repair [13].

The popularity of PARP inhibitors (PARPi) is based on several studies in breast and ovarian cancer showing that PARPi could selectively kill HR-deficient cancer cells [9, 10, 15]. This led to the FDA-approval of three PARP inhibitors for ovarian cancer, namely Olaparib, Rucaparib and recently Niraparib [17]. The precise mechanism of action remains a matter of active debate, although it is thought that the cytotoxicity to HR-deficient cells is due to the accumulation of SSBs in the absence of PARP1. This leads to replication fork collapse and DSBs, which need HR to be repaired [18, 19]. As BRCA1 and BRCA2 are essential for HR, these DSBs cannot be repaired, which will eventually lead to cell death [20]. Also PARP trapping contributes significantly to the synthetic lethality [21]. More mechanisms of action of PARP inhibitors are suggested, although these two are the most apparent and reported ones. These mechanisms show that the combination of HR-deficient cells with PARP1 inhibition is synthetically lethal [8, 13, 15].

### PARP inhibitors investigated in HNSCC

In the development of DNA damage response agents, PARPi have advanced the furthest. Several small molecule inhibitors are in clinical development: Niraparib, Talazoparib, Olaparib, Veliparib, Rucaparib and Iniparib [22]. Combining DDR inhibitors with DNA damaging agents has been the natural first step in combination strategies [21].

*In vitro* studies showed that HR-deficient HNSCC cells are hypersensitive to PARPi as they are unable to repair radiation-induced SSBs and that PAR induction by RT is probably prevented [18, 23–25]. Moreover, it is expected that PARPi would also work in HR-proficient cells since replication-dependent conversion of SSBs to DSBs focusses on rapidly proliferating cells more than on normal cells [22]. This hypothesis was confirmed in other studies where both HR-deficient and HR-proficient HNSCC cells were radiosensitized by Olaparib [24, 25]. As expected, lower concentrations were needed in HR-deficient cells to obtain the same radiosensitizing effect [22]. In the study of Weaver *et al*., HPV-positive cells had an increased sensitivity to PARPi as these cells were unable to recruit DNA-dependent protein kinase (DNA-PK) and BRCA2 to repair crosslinking damage [26]. These findings show HR-deficiency in HPV-positive cells as was earlier shown in the study of Dok *et al*. [27]. Recently, the idea was postulated to combine PARPi with HR inhibiting agents to broaden the applicability of PARPi. Possible agents to do so, are inhibitors of checkpoint kinase 1 (CHK1), WEE1, phosphatidylinositol-4,5-bisphosphate 3-kinase (PI3K), EGFR or heat shock protein 90 (HSP90) [28].

When PARPi were combined with CT and RT, potentiating effects were seen. The chemopotentiating effect of PARPi was demonstrated in oral cavity cancer cells as Cisplatin attenuates the repair of radiation-induced DSBs [7, 22, 29]. PARPi sensitizes cancer cells to platinum-based drugs, Temozolomide and topoisomerase poisons. These promising synergistic effects are further tested in multiple ongoing clinical trials, which combine PARPi with RT, CT or Cetuximab (see Table 1).

**Table 1:** Ongoing clinical trials evaluating PARP inhibitors in head and neck cancer patients

Compound	Combination	Phase	State	NCT number
***Olaparib***	/	1	Not yet recruiting	NCT02686008
	RT	1	Recruiting	NCT01460888
	RT	1	Recruiting	NCT02229656
	RT + Cisplatin	1	Recruiting	NCT02308072
	RT+ Cisplatin	1	Withdrawn	NCT01491139
	RT+ Cisplatin	1	Recruiting	NCT01562210
	Cisplatin	2	Recruiting	NCT02882308
	RT + Cetuximab	1	Recruiting	NCT01758731
***Veliparib***	Chemotherapy	1/2	Suspended	NCT01711541
***Talazoparib***	/	1	Not yet recruiting	NCT02567396

RT: Radiotherapy, NCT: Number clinicaltrials.gov identifier.

### PARP inhibitor biomarkers

BRCA1 and BRCA2 mutations are recognized as molecular targets for PARPi in several tumor types [11]. HR mutations rarely occur in HNSCC, with only 6% of the patients having BRCA1/2 mutations [30]. Other processes affecting HR might influence the cellular response to PARPi as there is evidence that a larger group of HNSCC is HR-deficient [18]. Alternative HR repair genes that sensitize cancer cells to the inhibition of PARP, often referred as 'BRCAness' have been identified [14]. Defects in the Fanconi anemia (FA) pathway and mutations in ataxia telangiectasia (ATM), ataxia telangiectasia and Rad3-related (ATR) or phosphatase and tensin homolog (PTEN) are suggested to contribute to the HR-deficient phenotype [10, 15, 19, 21, 31, 32]. Therefore, other accurate biomarkers than BRCA1 and BRCA2 are necessary to indicate HR-deficiency. Different HR-deficiency assays are available, but must be tested in prospective clinical trials. For example, the BROCA assay can identify mutations in 13 HR-genes via next generation sequencing. Secondly, functional assays can provide real-time information about DNA repair, like RAD51 focus formation assay for the activation of HR machinery and γH2AX foci to reflect DSBs, but cannot be implemented in clinical practice due to technical reasons [33].

Besides testing for HR-deficiency, multiple tests are available to predict PARPi response, such as loss of heterozygosity (LOH) which is gaining popularity as surrogate for HR and to predict PARPi response [34]. LOH is a large chromosomal event that results in the loss of an entire gene together with the surrounding chromosomal region. These genomic scars can be identified by the Myriad Genetics HR assay, which is now tested in prospective clinical trials in breast cancer [11]. According to Stover *et al*. LOH profiling might be more predictive for PARPi response than HR-deficiency assays in early disease stage [19]. Besides LOH, the combination of p53-binding protein 1 (53BP1) and BRCA1 was recently suggested as biomarker for PARPi sensitivity, as 53BP1-deficiency would impair sensitivity to PARPi [35]. Finally, PARP1 enzyme levels seem to be a logic biomarker for PARPi activity, but this does not correlate with the clinical response. So in conclusion, a variety of assays are available to assess HR-deficiency and to predict PARPi response although the optimal, clinically feasible assays need to be validated in clinical trials [11, 19, 21]. Two ongoing clinical trials in HNSCC have foreseen to do laboratory biomarker analyses (NCT02567396 and NCT01711541).

### Future challenges with PARP inhibitors

PARP inhibitors are the cornerstone of DNA repair targeted therapies [36]. The efficacy of PARP inhibition was demonstrated in BRCA1/2 mutated ovarian cancers. Although PARPi are well tolerated in ovarian cancer treatment, careful monitoring for long-term toxicity is mandatory [37]. Moreover, majority of patients will develop resistance eventually. Three mechanisms of resistance are identified so far. First, the HR function can be restored by secondary mutations in the BRCA1/2 gene together with loss of 53BP1 [9]. Secondly, an upregulation of polyglycoprotein 1 (PgP) pumps can cause efflux of the drugs. Finally, loss of PARP1 expression can lead to 100-fold resistance [14, 21]. Continued identification of resistance mechanisms is critical for further clinical use of PARPi.

HR-deficient HNSCC cells have shown hypersensitivity to PARPi, although the applicability of PARPi could be broadened to HR-proficient cells as Olaparib showed radiosensitization in both. These promising preclinical results are translated in ten ongoing clinical trials in the HNSCC field. Challenges for PARP inhibitor research remain to optimize the clinical efficacy and widen the utility of PARPi as most preclinical work is now focused on HR-deficient cells, leaving the question how PARPi sensitizes HR-proficient HNSCC cells unanswered [22, 38]. Furthermore, combination schedules with PARPi need to be further investigated where concomitant induced toxicities and pharmacological drug-drug interactions are important challenges. Therefore, optimization of the dose regimen and sequence becomes the key factor in successful clinical trial design, accompanied by accurate biomarkers that enable patient selection [14, 21, 22].

### PIKK family

The phosphatidylinositol 3-kinase-related kinases (PIKK) family is a serine/threonine kinase family that consists of six members: DNA-PK, PI3K, mechanistic target of rapamycin (mTOR), ATR, ATM and human suppressor of morphogenesis in genitalia-1 (hSMG-1). Family members DNA-PK, ATM and ATR play an important role in DDR, along with PI3K and mTOR that are involved in cell growth and differentiation. Due to the conserved kinase domain, inhibitors are often active against multiple members of the PIKK family [39].

### DNA-PK

DNA-PK has an extensive role in tumor associated processes including: G2/M cell cycle checkpoint regulation, genomic stability, hypoxia, metabolism, transcription support and inflammation making its inhibition an attracting therapeutic target [29, 40–42]. However, the established role of DNA-PK in innate immunity and pro-inflammatory signaling is an important aspect to consider with respect to long-term DNA-PK inhibitor use [21]. Furthermore, DNA-PK is an essential component of the NHEJ pathway, where it mediates direct ligation of broken DSBs and recruits other repair molecules [19, 21, 43, 44]. The precise mechanism how DNA-PK acts in NHEJ is beyond the scope of this review but is clearly described in the following articles [9, 21, 42–44]. In addition, there is evidence for a role of DNA-PK in HR. Though it is not definitively determined if DNA-PK promotes HR or if the failure of NHEJ promotes HR [42]. The pharmacological inhibition of DNA-PK results in inefficient repair and hypersensitivity to DSBs, hence suggesting its susceptibility to DNA-damaging agents.

### DNA-PK inhibitors

Different DNA-PK inhibiting molecules target the ATP binding site of the kinase. Early inhibitors are Wortmannin and LY294002, which are both non-specific DNA-PK inhibitors that showed *in vivo* toxicity and off-target effects resulting in a narrow therapeutic index [21]. Modifications of LY294002 led to two highly specific molecules, NU7441 and NU7026, both showing promising preclinical results as chemo- and radiosensitizers. However, their poor water solubility and oral bioavailability must be taken into account in further clinical evaluation. These problems are addressed in KU0060648, a dual DNA-PK and PI3K inhibitor with a better oral bioavailability and pharmacokinetic profile. Other DNA-PK inhibitors under investigation are: CC-122 a pleotropic pathway modifier, CC-115 a DNA-PK and mTOR inhibitor, VX-984 and MSC2490484A.

Remarkably, all agents are focused on the kinase subunit of DNA-PK, but the inhibition of the regulatory Ku subunit could also reduce DNA-PK activity [40]. Other approaches for DNA-PK inhibition could be nucleotide or antibody based inhibitors, which showed to have significant effects *in vitro* [44]. These could overcome the two primary faced obstacles with DNA-PK inhibitory compounds, namely poor water solubility and short serum half-lives [44]. The development of new DNA-PK inhibitors with good ADME (absorption, distribution, metabolism and elimination) profiles will be based on the recently discovered X-ray crystal structure of DNA-PK [40, 44].

### DNA-PK inhibitors investigated in HNSCC

Monotherapy with DNA-PK inhibitors has modest effects, but there is potential for antitumor synergy in combination with DNA-damaging agents [21]. Cells defective in DNA-PK are highly sensitive to RT, indicating that DNA-PK inhibition could be radiosensitizing [7]. This hypothesis was confirmed in different preclinical studies and was attributed to the fact that NHEJ is the primary pathway for the resolution of radiation-induced DSBs [26, 44]. Inhibition of DNA-PK promotes radiation-induced cell killing via mitotic catastrophe, senescence and autophagic cell death. Both NU7026 and NU7441 are proven to sensitize topoisomerase 2 inhibitors and are extreme radiosensitizers [45, 46]. Moreover, the radiosensitizing effect of NU7411 was shown in multiple cancer types: lung cancer cells, liver cells and breast cancer cells due to increased G2/M accumulation and prolonged delay in radiation-induced DSB repair [15, 41, 46–49]. The radiosensitizing effect is further increased in EGFR overexpressing cells as EGFR normally promotes NHEJ via DNA-PK [8, 50, 51]. Therefore, the effect of combining Cetuximab with DNA-PK inhibitors would be an interesting research topic.

The promising chemopotentiating and radiosensitizing effects of DNA-PK inhibitors are translated in multiple ongoing clinical trials in solid tumors, although none are listed in HNSCC specifically (see Table 2). CC-115 was well tolerated in a phase 1 trial with preliminary antitumor effects [21]. These promising *in vitro* results suggest it would be interesting to combine CC-115 with platinum-based chemotherapy in HR-deficient tumors [9].

**Table 2:** Ongoing clinical trials with DNA-PK inhibitors in solid tumors

Compound	Subject	Phase	State	NCT number
***CC-115***	Advanced solid tumors	1	Not yet recruiting	NCT01353625
***MSC2490484A+RT***	Advanced solid tumors	1	Recruiting	NCT02316197
	Solid tumors	1	Recruiting	NCT02516813
***C-122***	Advanced solid tumors	1	Recruiting	NCT01421524
***VX-984***	Healthy volunteers	1	Recruiting	NCT02644278

RT: Radiotherapy, NCT: Number clinicaltrials.gov identifier.

### DNA-PK inhibitor biomarkers

The DNA-PK inhibitor KU0060648 showed synthetic lethality in ATM-defective cells [9]. It has also been proven in the study of Shaheen *et al*. that ATM-deficient cells are addicted to DNA-PK for survival [15]. This suggests that HR-deficiency in general is associated with NHEJ addiction [9]. Mutations in HR genes (BRCA1/2, RAD51 and ATM) can predict sensitivity to KU0060648. These genes could be used as biomarker, but this has not been studied sufficiently. Biomarkers that are already used in preclinical research are similar as for the PARPi: γH2AX and RAD51 foci [52]. Biomarkers for NHEJ inhibitors are not yet validated [19].

### Future challenges with DNA-PK inhibitors

The radiosensitizing effect of DNA-PK inhibitors is proven in multiple cancer types, even though the effect as monotherapy is small. Unfortunately research in the HNSCC field is confined and there is only one clinical trial ongoing for the moment (NCT02516813). However, HNSCC could be a very attractive target for the application of DNA-PK inhibitors as p53 is often mutated or deregulated by HPV and HR-deficient cells are addicted to NHEJ, so synthetic lethality can be induced [19, 26]. Since KU0060648 efficiently radiosensitized ATM-deficient cells and these cells are addicted to DNA-PK for survival, it can be a promising approach to combine PARPi with DNA-PK inhibitors. Moreover, there appears to be a relationship between PARP and DNA-PK as PARP inhibited cells are susceptible to DNA-PK inhibition via the alternative NHEJ pathway [16, 50]. In comparison with PARPi, biomarkers for DNA-PK inhibitors are limited to HR-deficiency assays and are not validated for NHEJ inhibition. These two gaps in the area of DNA-PK inhibitory agents encourage research in the HNSCC domain as many promising results are awaited.

### PI3K inhibitors

The PI3K/AKT/mTOR pathway is heavily implicated in tumorigenic processes such as proliferation, invasion, apoptotic resistance, angiogenesis and metastases [53, 54]. The PI3K pathway, which is downstream of EGFR, is activated by growth factors and cytokines [55]. Important players in this pathway are protein kinase B (AKT), a second messenger, and the central mTOR complex which will promote cell growth via downstream effectors. This cascade is negatively regulated by the tumor suppressor gene PTEN. The RAS/RAF/MEK/ERK pathway is closely linked to the PI3K/AKT/mTOR pathway as there is crosstalk between PI3K and RAS [56, 57]. The precise mechanism of the pathway is beyond the scope of this review, but is discussed in the following articles [53, 54].

The PI3K/AKT/mTOR pathway is the most frequently deregulated pathway in HNSCC both on genomic and proteomic level [54]. Activating mutations of PI3K catalytic subunit 110α (PIK3CA) are seen in 56% of HPV-positive HNSCC and 39% of HPV-negative HNSCC according to The Cancer Genome Atlas (TCGA) [58–60]. Other deregulating mutations are possible in the regulatory subunit of PI3K, p85, which lead to activation of the PI3K pathway without growth factor stimulation. Furthermore, mutations in PTEN, AKT and the mTOR complex can result in the constitutive activation of the pathway leading to cell growth and proliferation in the absence of nutrients. Activation of this pathway is an important mechanism in resistance to EGFR inhibitors and cRT promoting tumor progression [55].

### PI3K inhibitors investigated in HNSCC

Therapeutic agents can tackle this pathway and promising *in vitro* and *in vivo* results show less proliferation, more apoptosis and sensitization to therapy. However, PI3K inhibition alone can trigger compensatory feedback via the RAS/MEK/ERK pathway or EGFR which induces resistance. Combination therapy with other therapeutic agents or DNA damaging agents can achieve synergistic effects [54, 61, 62]. RT activates EGFR and other prosurvival pathways like PI3K. When PI3K is inhibited, this causes downregulation of BRCA1/2, which are important in HR. Eventually this leads to inhibition of radiation-induced DDR [22]. Different types of PI3K inhibitors are developed and tested in preclinical research.

### PAN-PI3K inhibitors

Pan-PI3K agents inhibit more than one isoform of PI3K and are directed to tumors with PIK3CA mutations that are addicted to the PI3K pathway for growth and survival. Currently used inhibitors in clinical trials for HNSCC are Buparlisib and Copanlisib.

Buparlisib, also known as BKM120, is an oral reversible PI3K inhibitor competing to ATP [63, 64]. The anti-proliferative and pro-apoptotic effects of BKM120 are proven in tumor cells, irrespective the PIK3CA status [65]. However the half maximum inhibitory concentration (IC_50_) of BKM120 to fully block all PI3K forms as monotherapy is high, leading to cellular toxicity via tubulin [66]. The combination of BKM120 with Cetuximab showed synergistic effects in PIK3CA-mutant and wildtype HNSCC cell lines via the downregulation of AKT and induces apoptosis when BKM120 was given after Cetuximab [54, 67]. When these drugs are given in the opposite order an antagonistic effect is achieved since BKM120 downregulates the PI3K/AKT/mTOR pathway. In addition cells probably activate other pathways like the RAS/RAF/MEK/ERK pathway to rescue cell survival leading to treatment failure. These synergistic results were tested *in vivo* by Bozec *et al*. in an orthotopic HNSCC model [58]. Here, the synergistic effect of BKM120 with Cetuximab was seen as a significant inhibition of tumor growth, Ki67 reduction and mitogen-activated protein kinase (MAPK) pathway inhibition. Interestingly, RT alone normally upregulates the MAPK pathway as a compensatory mechanism and is implicated in tumor repopulation. This repopulation is a major challenge in the treatment of cancers but strikingly, the triple combination of RT with Cetuximab and BKM120 suppresses this radiation-induced activation of the MAPK pathway and combines anti-proliferative with pro-apoptotic effects [58].

The combination of BKM120 with Cetuximab is tested in multiple ongoing clinical trials (see Table 3). Results of a phase 2 study combining BKM120 with Paclitaxel showed improved clinical efficacy with a manageable safety profile, indicating an effective second-line treatment for metastatic HNSCC [68].

**Table 3:** Ongoing clinical trials with PAN-PI3K inhibitors in head and neck cancer patients

Compound	Combination	Phase	State	NCT number
***Buparlisib***	/	2	Unknown	NCT01527877
	/	2	Recruiting	NCT01737450
	Cisplatin + IMRT	1b	Recruiting	NCT02113878
	Paclitaxel	2	Not yet recruiting	NCT01852292
	Cetuximab	2	Not yet recruiting	NCT01816984
	Cisplatin/ Carboplatin	1b	Not yet recruiting	NCT02439489
***Copanlisib***	Cetuximab	1+2	Recruiting	NCT02822482

IMRT: Intensity-modulated radiation therapy, NCT: Number clinicaltrials.gov identifier.

Copanlisib is another highly selective and potent PI3K inhibitor with superior antitumor activity in PIK3CA mutants. The combination of Copanlisib with Cetuximab is now evaluated in a phase 1 and 2 trial in metastatic HNSCC patients with PIK3CA mutations or amplifications and/or PTEN loss as PIK3CA mutations and amplifications are associated with resistance to Cetuximab therapy (see Table 3) [69]. Copanlisib showed good tolerability and a dose-dependent pharmacokinetic in a recent phase 1 trial in Japanese patients [70].

### PI3K selective inhibitors

In contrast to pan-PI3K inhibitors, selective PI3K inhibitors focus on one specific isoform of PI3K. Mostly the 110α isoform, as this is the most commonly mutated component of PI3K [54]. Selective PI3K inhibitors are expected to produce more targeted inhibition with fewer side effects [59].

Alpelisib, also known as BYL-719, is one of the most promising selective PI3K inhibitors with a favorable safety profile. HNSCC cells proved to be sensitive and *in vivo* experiments showed dose-dependent growth inhibition in PIK3CA-mutated xenografts, indicating a superior effect in tumors with PIK3CA mutations [71]. However, synergistic effects were demonstrated *in vitro* and *in vivo* combining Alpelisib with Cetuximab, irrespective of the PIK3CA mutational status (69). Thanks to the encouraging preclinical results, 6 clinical trials are now ongoing (see Table 4) [54]. Early study results combining Alpelisib with Cetuximab showed promising antitumor activity in patients with recurrent HNSCC [69].

**Table 4:** Ongoing clinical trials in head and neck cancer with selective PI3K inhibitors

Compound	Combination	Phase	State	NCT number
***Alpelisib***	Cisplatin + IMRT	1	Recruiting	NCT02537223
	Paclitaxel	1	Completed	NCT02051751
	Cetuximab + IMRT	1	Recruiting	NCT02282371
	Cetuximab	1b	Terminated	NCT01602315
	/	2	Not yet recruiting	NCT02145312
	Cetuximab + Cisplatin	1/2	Not yet recruiting	NCT02298595
***AMG319***	/	2	Recruiting	NCT02540928

IMRT: Intensity-modulated radiation therapy, NCT: Number clinicaltrials.gov identifier.

Other selective PI3K inhibitors are on the market as well: INK1117, CAL-101 and AMG319. Cal101 and amg719 target the δ subtype, which is involved in regulatory T cells, with the purpose to break tumor-induced immune tolerance. When 110δ is inactivated by AMG319, CD8+ cytotoxic T cells are unleashed to induce tumor regression [54]. Currently, AMG319 is tested in a phase 2a trial in HPV-negative HNSCC patients (see Table 4) [54, 59]. CAL-101, also known as Idelalisib, has been approved by the FDA and the European Medicines Agency as the first-in-class PI3K inhibitor for hematological cancer therapy [72].

### AKT inhibitors

The phosphorylation of AKT is a very important step in the PI3K pathway and therefore many AKT inhibitors have been developed for cancer treatment. Together with the fact that AKT activation and overexpression is often associated with chemo- or radiotherapy resistance, these drugs can have great potential in cancer treatment [54]. Two types of AKT inhibitors are possible, an ATP-competitive variant such as GSK690693 and an allosteric inhibitor represented by MK-2206. MK-2206 has proven to be highly potent in enhancing the activity of anticancer agents *in vitro* and *in vivo* [54]. Very promising results came out of two clinical trials with MK-2206 in HNSCC patients (Table 5). In a phase 1 trial two HNSCC patients showed complete and partial response and in a phase 2 study with 21 patients, 9 patients were alive and progression-free at the end of the trial [73].

**Table 5:** Clinical trials with the AKT inhibitor MK-2206 in head and neck cancer patients

Compound	Combination	Phase	State	NCT number
***MK-2206***	/	2	Completed	NCT01349933
	/	2	Completed	NCT01370070

NCT: Number clinicaltrials.gov identifier.

### mTOR inhibitors

Other possibilities to attack this pathway are by focusing on the central mTOR complex. Most mTOR inhibitors are derived from Rapamycin, also known as rapalogues, for example Temsirolimus and Everolimus. Rapalogues bind to mTORC1 and stereotacticly interrupt the ability to signal to the downstream effectors [53, 54]. Temsirolimus is already approved by the FDA to treat renal cell carcinoma. In contrast to its analogue Everolimus, Temsirolimus showed very promising results in a phase 2 trial in HNSCC [58, 74, 75]. In an orthotopic HNSCC model, the combination of Temsirolimus with Cetuximab inhibited the PI3K pathway as well as the MAPK pathway, and showed antiangiogenic effects, leading to almost a complete tumor response [58]. Multiple clinical trials in HNSCC patients with Temsirolimus are completed and indicated a significant inhibition of mTOR (see Table 6) [76]. In a trial with platinum- and Cetuximab-recurrent HNSCC patients, Temsirolimus treatment induced stabilization in 58% of the patients and 39% showed tumor shrinkage. However, combination treatment with Temsirolimus can be limited due to numerous toxicities and overall the therapeutic benefit was unsatisfactory [54, 66, 77].

**Table 6:** Clinical trials with Temsirolimus in head and neck cancer patients

Compound	Combination	Phase	State	NCT number
***Temsirolimus***	Paclitaxel + Carboplatin	1/2	Not yet recruiting	NCT01016769
	/	2	Completed	NCT01172769
	/	unknown	Completed	NCT00195299
	Cetuximab	2	Completed	NCT01256385
	Erlotinib	2	Completed	NCT01009203
	Cetuximab + Cisplatin + RT	Pilot	Withdrawn	NCT01326468
	Cetuximab + Cisplatin	1/2	Terminated	NCT01015664

RT: Radiotherapy, NCT: Number clinicaltrials.gov identifier.

The effect of mTOR inhibition could even be broadened via dual pan-class 1 PI3K-mTOR inhibitors that are based on the structural similarities of the catalytic subunit of mTOR and the p110 subunit of PI3K. Thanks to this, the pathway is targeted at two levels. Examples of dual inhibitors are SF1126, PF-05212384, BEZ235, GSK1059615 and XL765. These dual inhibitors all showed radiosensitizing effects in HNSCC cells and multiple clinical trials are ongoing in the HNSCC field (see Table 7) [54, 59, 78–81]. These dual inhibitors are not discussed in detail as this is beyond the scope of this review, but can be explored in the following articles [79, 82].

**Table 7:** Clinical trials with dual PI3K-mTOR inhibitors in head and neck cancer patients

Compound	Combination	Phase	State	NCT number
***PF-05212384***	PD-0332991	1	Recruiting	NCT03065062
	Paclitaxel, Carboplatin	1	Recruiting	NCT02069158
***SF1126***	/	2	Recruiting	NCT02644122
***BEZ235***	/	1	Completed	NCT00620594

NCT: Number clinicaltrials.gov identifier.

### Biomarkers

To assess the level of inhibition of the PI3K pathway, the phosphorylation level of AKT or its downstream effectors gives a good indication [61, 79]. The phosphorylation status of AKT was significantly associated with the sensitivity for the dual PI3K-mTOR inhibitor PF-05212384 in HNSCC cells and can predict resistance to standard HNSCC therapies like Cetuximab and RT, regardless the PIK3CA status. Even better, this resistance can be overcome by combination of mTOR-PI3K and MEK inhibition therapy [57]. The major drawbacks to use AKT phosphorylation level as biomarker are the strict handling and sampling conditions of the tissue, which makes implementation in clinical trials difficult [59, 61].

Preclinical studies indicated that tumors with PIK3CA mutations were more sensitive to PI3K inhibition treatment in contrast to PTEN loss which indicated resistance [58, 60]. Early clinical trials have not found a clear correlation between molecular alterations in the PI3K pathway and antitumor effects of the therapy. However tumors with activating mutations in members of the MAPK pathway are potentially resistant to PI3K inhibitors [58, 59]. These conflicting results make the predictive value of PIK3CA or PTEN mutations inconclusive and unable to implement in clinical practice. However, these contrasting findings can be attributed to external factors. First, PI3K alterations can be missed with early detection methods that were based on a limited number of assays or the lack of well-defined thresholds to define PTEN loss for example. Secondly, other alterations in genes such as AKT1/2 encoding AKT, PIK3R1 encoding the regulatory subunit p85α, liver kinase B1 (LKB1) that activates AMPK and neurofibromin 1 (NF1) that encodes the negative regulator of the RAS pathway, can induce sensitivity. Furthermore, PI3K-mutant HNSCC patients could also have coexisting mutations that induce resistance to PI3K inhibitors, such as KRAS mutations. Tumor heterogeneity and incorrect or invalidated assays can also explain the variation. These tumor-based markers should be expanded so that not only genomic aberrations in PIK3CA, PTEN, ATKT1/2, etc. could be targeted, but also a ‘PI3Kness’ status is included that can serve as indicator for all activating alterations. Finally, a recent study concluded that 15% of the mutations in PI3K pathway genes are subclonal rather than truncal. These subclonal driver mutations can explain the uncertain predictive value of PI3K/AKT/mTOR mutations and suggest that the response to PI3K inhibitors should be assessed by the proportion of tumor cells in which the driver mutation is identified [61].

The complexity of the pathway and its feedback loops hypothesizes that a clear prediction of the response via genotype will be difficult. Furthermore, PI3K inhibitors can have a plethora of effects on tumors, going from angiogenesis to immune cells and other environmental interactions. Therefore it is probable that not a single biomarker can predict sensitivity but rather a molecular signature will be required [61].

### Future challenges of PI3K inhibitors

PI3K/AKT/mTOR inhibitors can effectively inhibit the PI3K pathway and are well tolerated [59]. Preclinical research with PI3K inhibitors in HNSCC has provided very promising results. Translation to clinical trials was expected to have encouraging results, which is unfortunately not the case. Different explanations could address this discrepancy. As discussed previously, PI3K inhibitors as monotherapy have limited effect due to the compensatory feedback via other pathways like RAS/RAF/MEK/ERK. This can be addressed by combining PI3K inhibitors with DNA damaging therapies or Cetuximab, which results in dramatic growth inhibition via an anti-proliferative effect rather than a pro-death action [80]. Secondly, PI3K inhibition can induce mitochondrial reprogramming that will promote tumor invasion and progression. Furthermore, preclinical research that provides optimal dosing schedules, is scarce although this is very important for clinical trial success. Most of all, inadequate patient selection can affect trial results. The correlation between PI3K pathway mutational status and responsiveness is unclear and currently patients are selected based on prior treatment failure. This lack of patient stratification can have tremendous effects on response to targeted therapies. Evidently there is room for improvement, combining PI3K inhibitors with other DNA damaging therapies or Cetuximab may have clinical benefit regardless of the mutational status [80]. To improve the success of PI3K inhibitors, pharmacological, biological and translational issues must be better identified. Valuable biomarkers, which are necessary to correctly use these targeted agents, need to be determined, but it is likely that this will depend on the tumor type, genotype and the compound. Biomarker-driven clinical trials are necessary to answer these questions together with thorough preclinical research to stratify patients and predict response [54, 61].

### ATM and ATR inhibitors

The cell cycle consists of different checkpoints that are activated in case of DNA damage to ensure genomic stability [83]. The G2/M checkpoint is the last major opportunity to prevent that DNA damage is taken into mitosis which otherwise leads to mitotic catastrophe and cell death [84]. ATM and ATR are two critical components of the DDR that sense DNA damage to activate the G2/M cell cycle checkpoint. They are both members of the PIKK family, making it difficult to design specific inhibitors [83]. ATR and ATM phosphorylate CHK1 and CHK2 respectively to eventually arrest the cell cycle enabling DDR. Somatic mutations in ATR and ATM are seen in various frequencies in HNSCC with 4–10% and 1–16% for ATR and ATM respectively [30].

### ATM and ATR inhibitors investigated in HNSCC

Three ATR and ATM inhibitors have already been developed: VX-970 and AZD6738 as ATR inhibitor and AZD0156 as ATM inhibitor. VX-970, previously known as VE-822, is the first-in-class ATR inhibitor which has a sensitizing effect to chemotherapeutic drugs that induce replication fork collapse like Cisplatin. VX-970 increased the antitumor activity of Cisplatin *in vivo* in patient derived xenograft models which is translated to an ongoing phase 1 trial in HPV-negative HNSCC (see Table 8) [19, 51]. A phase 1 trial with VX-970 as monotherapy showed good tolerability. Secondly, AZD6738 is another potent, selective, oral ATR inhibitor that demonstrated radiosensitization in a panel of human cancer cell lines independent of the p53 or BRCA2 status and is currently tested in phase 1 clinical trials as monotherapy or in combination with radiotherapy, chemotherapy or Olaparib [19, 21, 85]. Two clinical trials are currently ongoing with AZD6738 combined with Olaparib in HNSCC and these are investigating possible biomarkers (Table 8).

**Table 8:** Clinical trials with ATR inhibitors in HNSCC patients

Compound	Combination	Phase	State	NCT number
***AZD6738***	Olaparib, Carboplatin or MED14736	1	Recruiting	NCT02264678
	Olaparib	1	Recruiting	NCT03022409
***VX-970***	Cisplatin, RT	1	Recruiting	NCT02567422

RT: Radiotherapy, NCT: Number clinicaltrials.gov identifier.

### Biomarkers

Currently, biomarkers in ATM and ATR inhibitors are limited. Alterations in ATM/ATR and DDR deficiencies are suggested to increase reliance to cell cycle checkpoints. Also alterations that cause increased replication stress can be possible biomarkers, such as p53 mutations, Cyclin E upregulation and RAS or MYC mutations. A more advanced biomarker is alternative lengthening of telomeres (ALT), together with loss of ATP-dependent helicase (ATRX), as the loss of ATRX results in increased ALT. Therefore, the loss of ATRX and the increase in ALT may predict the response to ATR inhibitors [19]. In a study in HNSCC combining ATR and CHK1 inhibitors evoked distal loss of 11q as a possible biomarker [86]. Since research with ATM inhibitors is confined, the only suggested biomarker is H2AX foci as for other DNA repair inhibitors.

### Future challenges with ATR/ATM inhibitors

ATR inhibitors VX-970 and AZD6738 showed promising preclinical results which led to ongoing clinical trials in combination with other antitumor agents. Unfortunately, with the AZD6738 inhibitor it is a tradeoff between antitumor activity and bone marrow toxicity [21]. These toxicity issues should be addressed by thorough preclinical research focused on optimal scheduling of the combined agents. To select proper HNSCC patients, the above suggested biomarkers must be validated so that profound clinical trials can be designed in the future. Furthermore, ATR inhibition activates backup pathways via DNA-PK and CHK1 as the ATR-CHK1 axis is not linear [84]. This could be circumvented by combining ATR inhibition with DNA-PK or CHK1 inhibition, which is a possible novel strategy in HNSCC research.

### Cell cycle checkpoint targeted molecules

As mentioned before, the targets of ATR and ATM are the kinases CHK 1 and CHK2. After ATR activated CHK1, this will then phosphorylate CDC25. Additionally, CHK1 also activates WEE1 to phosphorylate the cyclin dependent 1 (CDK1)/CyclinB complex. The phosphorylation of CDK1 will activate the G2 cell cycle checkpoint and arrests the cell cycle so that the DNA damage can be repaired. This is normally prevented by the phosphatase CDC25 that dephosphorylates CDK1 to deactivate the checkpoint resulting in continuation of the cell cycle. The key components of the G2/M checkpoint, being CHK 1&2, WEE1 and CDC25, are interesting targets for cancer therapy as the activation of this checkpoint leads to DNA repair [6, 8, 22, 87].

### CHK1/2 inhibitors

As described above, CHK1 mediates the S- and G2 phase checkpoint and its function is important in DNA replication, cell cycle progression and survival [22, 40]. Furthermore, CHK1 promotes the recruitment of RAD51, involved in HR, to repair the DNA damage in the S phase [22, 83]. CHK2 promotes the G1 checkpoint regulated by p53. When p53 is mutated, the G1 checkpoint is abrogated. Therefore, p53-mutant cells rely more on the G2 checkpoint. Tp53 is mutated in 85% of HNSCC and additionally HPV causes the deregulation of p53 and retinoblastoma (pRb) via oncoproteins E6 and E7 [88, 89]. These cells are no longer protected by the G1 checkpoint, so when the S-G2 checkpoint would be abrogated by the inhibition of CHK1 and/or WEE1, the cell cycle would proceed despite the presence of DNA-damage which would lead to mitotic catastrophe and cell death [8]. Likewise, it would enhance apoptosis induced by DNA damaging agents. In contrast, p53-potent cells are protected by the intact G1 checkpoint which will stop the cell cycle and induce NHEJ [22]. Several studies have proven the sensitizing effect of CHK1/2 inhibitors to DNA damaging agents in p53-deficient cells [3, 6, 88]. The greatest potentiating effect was seen with antimetabolites like Gemcitabine, as antimetabolites temporarily redistribute cells into the S phase. This synchronization of cells maximizes the effect of CHK1 inhibitors on radiation-induced DSBs [22].

Different CHK1/2 inhibitors are already developed with UNC-01 and AZD7762 being the first that proved synergism with DNA damaging agents in humans [15]. However, lack of efficacy, pharmacokinetic and toxicity issues were revealed in early clinical trials [40, 45, 87]. LY2606368 is a strong ATP-competitive CHK1/2 inhibitor, being the improved analogue of LY2603618 that showed cardiotoxicity in phase 1 trial [40]. LY2606368 showed growth inhibition, but the poor oral bioavailability and intravenous administration are important disadvantages [84]. Next, MK-8776 also known as SCH-900776, showed good *in vitro* results and phase 1 trials combining MK-8776 with Gemcitabine showed clinical efficacy and moderate tolerability [21]. Other CHK1 inhibitors that recently entered clinical trials are: PF0477736, CCT245737, V158411, GDC-0425 and GDC-0575 [19, 21, 40, 83, 84].

### CHK1/2 inhibitors investigated in HNSCC

Preclinical studies in HNSCC confirmed the radiosensitizing effect of CHK1 inhibitors [22, 23, 87]. When PF0477736, AZD7762 or SAR-020106 is administered to p53-deficient cells, G2 arrest is abrogated which results in radiosensitization [90]. CHK1 inhibitors can be combined with PARP inhibitors to further enhance the radiosensitizing effect in HPV-positive cells [23]. Combining CHK1/2 inhibitors with Cetuximab and RT results in a significant delay of *in vivo* tumor growth without increased toxicity [91]. These promising effects of CHK1 inhibitors resulted in numerous clinical trials that are conducted in solid tumors with LY2603618, AZD7762 and CCT245737 as monotherapy or in combination with other DNA damaging therapies [11, 45]. Focusing on HNSCC, two clinical trials are ongoing with CHK1 inhibitors. LY2606368, or Prexasertib, is investigated as monotherapy in a completed phase 1 trial (NCT01115790) and is combined with Cisplatin and Cetuximab in an ongoing phase 1 trial in advanced HNSCC (NCT02555644) (see Table 9).

**Table 9:** Clinical trials with cell cycle checkpoint inhibitors in HNSCC

Class	Compound	Combination	Phase	State	NCT number
CHK1/2 inhibitor	LY2606368	/	1	Completed	NCT01115790
		Cisplatin, RT, Cetuximab	1	Recruiting	NCT02555644
WEE1 inhibitor	AZD1775	Cisplatin, RT	1	Recruiting	NCT02585973
		Cisplatin, RT	1	Not yet recruiting	NCT03028766
		Cisplatin, Docetaxel, Surgery	1	Recruiting	NCT02508246
		Cisplatin	2	Terminated	NCT02196168

RT: Radiotherapy, NCT: Number clinicaltrials.gov identifier.

### Biomarkers

CHK1 overexpression is the most suggested biomarker for CHK1 inhibition associated with sensitivity [19, 40, 83]. Some clinical trials use p53 status together with pCDK1/2 status as CHK1 pathway biomarker [92]. Other studies suggest that high levels of DNA-PK may be a potential biomarker to stratify patients to CHK1 inhibitor therapy, as cells deficient in DNA-PK were resistant to CHK1 inhibition [93].

### Future challenges of CHK1/2 inhibitors

CHK1 inhibitors showed radiosensitizing effects in p53-mutant and HPV-positive HNSCC cells. The combination of CHK1 inhibitors with Cetuximab and radiation induced significant tumor growth delay, which led to four ongoing clinical trials. However, the caveat when combining CHK1/2 inhibitors with DNA damaging agents is the schedule of administration as CHK1 is especially important for the stability of stalled replication forks, often caused by chemotherapy [15]. Chemotherapy should be given first, so that the cells accumulate in the S phase, then CHK inhibitors can be given and thereafter radiotherapy to maximize the effect [22].

### WEE1 inhibitors

WEE1 is responsible for the inhibitory phosphate on CDK1 and G2/M checkpoint activation [11]. Inhibition of WEE1 results in the abrogation of the G2 checkpoint and premature enter of mitosis [84, 94]. As with CHK1 inhibitors, p53-proficient cells are protected from G2 checkpoint abrogation due to the intact G1 checkpoint. The first-in-class small molecule WEE1 inhibitor AZD1775, also known as MK-1775, has shown to potentiate multiple chemo- and radiotherapies [11]. The IC_50_ of AZD1775 is 20 times lower in p53-mutated HNSCC cells than wildtype. Combining WEE1 inhibitors with DNA damaging agents has proven to be a rational strategy. When AZD1775 is combined with RT in HPV-positive cells, an increase in cell death is seen regardless the p53 status [88]. In combination with chemotherapy, this induces mitotic catastrophe and senescence in HPV-negative cells that are p53-mutated, whereas in HPV-positive cells it increases apoptosis [95].

Inhibition of WEE1 evokes an upregulation of CHK1 due to S phase accumulation and replication stress. This feedback mechanism reduces the efficacy of WEE1 inhibition, by combining CHK1 and WEE1 inhibitors this effect can be antagonized. Different studies have proven the synergistic effects of CHK1 and WEE1 inhibitors resulting in tumor growth reduction regardless the p53 status [88, 96]. Combining AZD1775 and LY2603618 showed more efficient radiosensitization with lower drug concentrations in HPV-positive cells while sparing normal cells [87]. Adding PARP inhibitors to the combination of WEE1 and CHK1 inhibitors increases the sensitivity to RT even further and is translated to a phase 2 clinical trial (NCT02576444) [28]. CHK1 and WEE1 inhibitors combined with RT and chemotherapy induced high levels of sensitivity due to G2 checkpoint and HR abrogation [22].

The promising chemo- and radiopotentiating effect of WEE1 inhibitors in HNSCC cells are translated to four ongoing clinical trials. Here, Cisplatin and RT are combined with AZD1775 while two other clinical trials are performing laboratory biomarker analyses next to the effect of the treatment (Table 9). Preliminary clinical trial results show good tolerability and minimal collateral cytotoxicity [95].

### Biomarkers

As indicated above, the status of p53 as biomarker remains a matter of active debate. The phase 1 trial that combined AZD1775 with chemotherapy showed superior results in p53-mutants in contrast to the phase 2 trial in non-small cell lung cancer that observed no association between p53 status and response [97, 98]. Further research is necessary to unravel the significance of the p53 mutational status on CHK1 and WEE1 inhibitors.

Possible biomarkers for combining AZD1775 with DNA damaging agents are not known yet. However, different studies are suggesting some markers like WEE1 and PAX-interacting protein 1 (PAXIP1) levels as mechanism-based biomarkers. PAXIP1 is a PAX protein essential for cells to progress through mitosis and regulates WEE1 activity [99]. Together with low PKMYT1 expression, a kinase functionally related to WEE1, this could serve as an enrichment biomarker for AZD1775 sensitivity [100]. Another study suggested WEE1 inhibition gene signature as pharmacodynamic biomarker based on mRNA expression in tissue biopsies [101]. According to a very recent study focused on the use of mitotic inhibitors in HNSCC, mutations in AJUBA, SMAD4 and RAS predict the sensitivity to CHK1 and WEE1 inhibitors [102]. However, these suggested biomarkers are not validated yet, nor implemented in clinical trials.

Partial response of a head and neck cancer patient with a BRCA1 mutation was seen in a phase 1 study, indicating that DNA repair pathways may be a therapeutic target in HNSCC patient selection [30]. A large-scale study in 59 HNSCC cell lines suggested that insulin receptor substrate 4 (IRS4) and SMAD4 mutations could predict sensitivity to CHK1/2 and WEE1 targeted agents [102]. As described in this article, HNSCC cell lines have a wide range of sensitivity indicating the need for thorough preclinical research to enable *in vivo* translation. Personalized treatment will be necessary for such specialized treatment strategies, therefore biomarkers are urgently awaited.

### Future challenges of WEE1 inhibitors

The combination of adduct-forming chemotherapies and radiotherapy with WEE1 inhibition is a reasonable strategy that should be further investigated in the future. For now the focus was laid on the combination with antimetabolites seen the synchronization of cells in the S phase, where WEE1 is most active. The combination schedule of CHK1 and WEE1 inhibitors with chemo- and radiotherapy is based on early preclinical studies, where a logical order of administration is the inhibitor given after chemotherapy but just before RT so that early repair is inhibited and sustained till extended time thereafter so that late repair and G2-S checkpoints are inhibited [22].

WEE1 inhibition forces the cells through the replication cycle resulting in genomic instability, apoptosis and mitotic catastrophe. A concern therefore is that cancer cells that survived this forced mitosis would increase their proliferation rate and become resistant to chemotherapy, as was seen in proliferation assays. Another important aspect for future research is to determine off-target effects of AZD1775 and improve the selectivity of WEE1 inhibitors [103].

Furthermore, CHK1 inhibitors combined with WEE1 inhibitors showed synergistic effects to result in growth reduction in HNSCC cells regardless the p53 status. Preclinical studies that investigate biomarkers are restricted, luckily ongoing clinical trials are making an attempt to do laboratory biomarkers analyses. However, biomarkers for the discussed inhibitors are unclear and invalidated, despite the fact that they are needed for appropriate patient selection. Another critical point in developing reasonable clinical trials, is the administration schedule. This should be based on the mechanism of action and must be tested in preclinical studies first.

### Conclusion

Combining DDR inhibitors with DNA damaging agents can overcome cancer defense mechanisms and improve therapy significantly. A plethora of preclinical studies proved the effect of DDR inhibitors in HNSCC. This review indicates that there is great potential for DNA repair targeted agents combined with antiproliferative therapies in HNSCC. This potential together with the high need for novel targeted therapies to improve the treatment of HNSCC stresses the need for good preclinical research to support rational clinical trial design.
